# Dentistry and Cranio Facial District: The Role of Biomimetics

**DOI:** 10.3390/biomimetics9070389

**Published:** 2024-06-26

**Authors:** Giuseppe Minervini

**Affiliations:** 1 Saveetha Dental College and Hospitals, Saveetha Institute of Medical and Technical Sciences (SIMATS), Saveetha University, Chennai 602105, Tamil Nadu, India; giuseppe.minervini@unicampania.it; 2 Multidisciplinary Department of Medical-Surgical and Dental Specialties, University of Campania, Luigi Vanvitelli, 80138 Naples, Italy

Biomimetics has emerged as a pivotal field, bridging fundamental research and practical applications. This area of research has aready had a significant impact across various industries and scientific disciplines [1,2]. This Special Issue presents a curated collection of pioneering research articles, highlighting the latest advancements and innovative approaches within the field. The contributions span diverse topics, showcasing the interdisciplinary nature and expanding influence of biomimetic research. Innovations in medical and dental technologies, enhanced by biomimetic principles, are driving the development of new techniques for improving implant success rates and designing advanced materials with superior properties [3,4,5]. Researchers are exploring the potential of natural substances to enhance the performance of synthetic materials and designing scaffolds that promote tissue regeneration [1,6]. These studies highlight how biomimetic approaches can lead to sustainable and efficient solutions, inspired by the principles observed in nature. Indeed, this Special Issue encapsulates the transformational possibility of biomimetics in developing innovations to solve the complex problems facing humanity today, opening a promising avenue for further research into the emerging gap between biological nature as an inspirational value and technological growth.

Herce-López et al. [7] conducted a comprehensive review and provided a consensus report on the use of customized subperiosteal implants (CSIs) to rehabilitate atrophic jaws. Their study was thus able to reveal that “the early survival rate of CSIs was high, and the incidence of major complications is considerably low”. Emphasis was placed on the observation that, while the biological and mechanical rates of complications ranged from 5.7% to 43.8% and 6.3% to 20%, respectively, the capacity to model these implants allowed for a better fit and lower morbidity than the traditional methods of bone grafting; hence, they represent a better choice for patients with bone that is deficient for traditional implantation. Custom-designed implants provide a better fit and, in comparison with the conventional bone grafting procedure, lead to less morbidity, thus offering great hope for alternative viability in patients with deficient bone who are not amenable to conventional implants [8,9]. The thorough digital planning required to ensure the passive fit of the CSI was highlighted as crucial to avoiding implant failure. The consensus report emphasizes that while CSIs are a promising treatment option for edentulous patients with atrophic jaws, available data remain scarce and additional research is needed to establish robust clinical guidelines [7,9,10,11,12,13].

Lee et al. [14] investigated the prophylactic effects of localized biomimetic minocycline and systemic amoxicillin on immediate implant placement in infected extraction sites. For the other groups tested, the study organized the twelve mongrel experimentees around six implants. These groups included the negative control, the infected control, the collection of samples infected and treated with amoxicillin, those others infected and treated with minocycline, and a group infected and treated with a combination of drugs. The findings indicated that systemic amoxicillin significantly improved implant success by preventing bone loss and reducing peri-implant soft tissue inflammation. Localized minocycline also reduced bone loss and increased removal torque (RT), a measure of implant stability. However, both antibiotics functioned as a combination that was not said to synergistically enhance the effect; in fact, it even showed some antagonistic activity, making this the likely interaction between amoxicillin and minocycline. This study underscores the importance of antibiotic choice and delivery method in improving implant outcomes, particularly in infected sites [14,15].

Alsunbul et al. [16] explored the reinforcing effects of gum Arabic (GA) on glass ionomer cements (GICs). In this experiential work, it was demonstrated that the addition of 0.5 wt.% GA into the GIC remarkably increased the flexural strength, fracture toughness, and tensile strength, but reduced the internal porosity. These enhancements were attributed to the effective dispersion of GA within the GIC matrix, which acted as a reinforcing agent. Interestingly, the addition of GA did not significantly affect the film thickness or water contact angle of the GIC formulations, indicating that modifications enhanced mechanical properties without compromising handling characteristics [17,18,19]. This outcome implies that GA reinforcement significantly increases the performance and durability of the GICs and, therefore, will find utility in a wide range of applications in dentistry [16].

In another study by Florea et al. [20], it was found that biomimetic hydroxyapatite toothpastes were very potent in inducing enamel remineralization and had the potential to prevent caries. Of the formulations tested, four of the experimental hydroxyapatite toothpastes showed that tetrasubstituted hydroxyapatite behaved not just optimally also with high potency. This formulation was most effective in enhancing the mineral density of enamel and preventing the progression of early tooth decay. Due to this reason, a large number of analytical techniques, including micro-CT and scanning electron microscopy, were applied to prove the ability to remineralize [21,22]. The overall results evidenced the effectiveness of the hydroxyapatite-based toothpaste for the treatment of initial caries and the improvement of oral health and suggested that it could be recommended for inclusion in everyday dental practice to prevent caries and strengthen enamel structures [20].

Valamvanos et al. [23] reviewed the latest developments in scaffolds for guided bone regeneration. They highlighted the design of biomimetic scaffolds with controlled features in which cell and tissue interaction can be modulated. They consist of a range of fabrication techniques, including but not limited to 3D printing, electrospinning, and solvent casting. Each of these offers a distinct advantage for the design of a scaffold [24]. This will open up an opportunity for the ability of these scaffolds to serve as growth factors and bioactive molecule delivery systems, something of very strong clinical relevance to bone repair and regeneration [25,26]. This article highlights, with promising preclinical work, that more research needs to be undertaken in order to translate these findings to the clinic. This review underscores the critical role of biomimetic scaffolds in advancing regenerative medicine and improving outcomes in bone repair [23].

A study by Alsaeed and Al-Ghaban [27] focused on the synergistic effects of chitosan nanoparticles (ChN) and simvastatin (Sim) on bone healing. The combination of ChN and Sim significantly enhanced bone formation and reduced osteoclastogenesis compared to controls. This was potentially due to the dual activity of chitosan as a biodegradable scaffold, along with simvastatin functioning to promote osteoblastic activity and inhibit osteoclast-mediated bone resorption. This study involved histological and histomorphometrical analyses to assess bone healing in experimental maxillary bony defects. The achieved results indicated that further efforts need to be directed at the association of biomaterials with pharmacologic agents to develop more effective interventions in bone-regenerating procedures. The integration of ChN and Sim offers a promising approach to enhancing bone healing, particularly in patients with compromised bone regeneration capacity [27,28,29].

Dias et al. [30] investigated the accuracy and reliability of two digital methods emplyoed for tooth color assessment: SpectroShade (SS) and eLAB. This study focused on comparing the Lab* values of VITA Classical (VC) and VITA Toothguide 3D-MASTER (VM) shade guides across different batches by employing these methods. The study concluded that while both SS and eLAB methods evidenced good internal consistency for tooth color assessment, they exhibited significant variability when compared with each other. This inter-device variability underscores the need for standardization and caution when interpreting color measurements across different devices. Clinicians should be aware of these differences to ensure accurate color matching in dental restorations [31,32]. These findings suggest that, despite the advancements in digital color assessment technologies, achieving consistent and reliable color matching remains challenging, necessitating ongoing efforts to improve these tools and their application in clinical practice [30,33,34].

The studies presented in this Special Issue underscore the transformative impact of biomimetics across various fields. With scientists beginning to employ nature-inspired principles, solutions are currently being devised which are not simply innovative, but also sustainable and effective. The success of this Issue of *Biomimetics* will pave the way for future lines of inquirty that will once again bridge the divide between biological inspiration and state-of-the-art technology.
